# Cryptic species and hidden ecological interactions of halictine bees along an elevational gradient

**DOI:** 10.1002/ece3.7605

**Published:** 2021-05-17

**Authors:** Antonia V. Mayr, Alexander Keller, Marcell K. Peters, Gudrun Grimmer, Beate Krischke, Mareen Geyer, Thomas Schmitt, Ingolf Steffan‐Dewenter

**Affiliations:** ^1^ Department of Animal Ecology and Tropical Biology Biocenter University of Würzburg Würzburg Germany; ^2^ Center for Computational and Theoretical Biology Campus Nord University of Würzburg Würzburg Germany; ^3^ Department of Bioinformatics Biocenter University of Würzburg Würzburg Germany

**Keywords:** COI, cuticular chemistry, elevational gradient, Halictidae, microbiome metabarcoding, pollen metabarcoding

## Abstract

Changes of abiotic and biotic conditions along elevational gradients represent serious challenges to organisms which may promote the turnover of species, traits and biotic interaction partners. Here, we used molecular methods to study cuticular hydrocarbon (CHC) profiles, biotic interactions and phylogenetic relationships of halictid bees of the genus *Lasioglossum* along a 2,900 m elevational gradient at Mt. Kilimanjaro, Tanzania. We detected a strong species turnover of morphologically indistinguishable taxa with phylogenetically clustered cryptic species at high elevations, changes in CHC profiles, pollen resource diversity, and a turnover in the gut and body surface microbiome of bees. At high elevations, increased proportions of saturated compounds in CHC profiles indicate physiological adaptations to prevent desiccation. More specialized diets with higher proportions of low‐quality Asteraceae pollen imply constraints in the availability of food resources. Interactive effects of climatic conditions on gut and surface microbiomes, CHC profiles, and pollen diet suggest complex feedbacks among abiotic conditions, ecological interactions, physiological adaptations, and phylogenetic constraints as drivers of halictid bee communities at Mt. Kilimanjaro.

## Graphical abstract

Changes of abiotic and biotic conditions along elevational gradients represent serious challenges to organisms which may promote the turnover of species, traits, and biotic interaction partners. We used molecular methods to study cuticular hydrocarbon (CHC) profiles, biotic interactions and phylogenetic relationships of halictid bees of the genus *Lasioglossum* along a 2,900 m elevational gradient at Mt. Kilimanjaro, Tanzania. The detected interactive effects of climatic conditions on gut and surface microbiomes, CHC profiles, and pollen diet suggest complex feedbacks among abiotic conditions, ecological interactions, physiological adaptations, and phylogenetic constraints as drivers of halictid bee communities at Mt. Kilimanjaro.

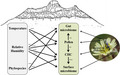

## 1 INTRODUCTION

Elevational gradients challenge all organisms as they are confronted with rapid abiotic and biotic changes over short geographic distances. These changes primarily, but not exclusively, involve abiotic shifts in temperature, humidity, precipitation, partial pressure of atmospheric gases, UV radiation, atmospheric turbulence, and wind speed (Barry, 1992). Consequences for organisms include reduced suitable time periods for development, foraging, and reproduction, but also changes in food quality and pressure by natural enemies and pathogens (Hodkinson, 2005). Several morphological, life history, and behavioral adaptations to the changing environment along elevational gradients have been investigated, such as changes in body, wing size, number of generations, or activity times (Classen et al., 2017; Hodkinson, 2005; Hoiss et al., 2012; Janzen, 1973; Janzen et al., 1976). Differing environmental conditions can even lead to changes in the nesting behavior of halictid bees from social to solitary nesting (Eickwort et al., 1996). But underlying physiological adaptations, ecological interactions, and phylogenetic constraints have never been linked within one study along elevational gradients.

Tropical mountains, such as Mt. Kilimanjaro, are ideal places to study changes of traits and interactions of animals across broad climatic gradients at feasible spatial scales (Sanders & Rahbek, 2012). Pollinators, especially bees, have key functions in ecosystems and are threatened by a variety of anthropogenic factors (Vanbergen & the Insect Pollinators Initiative, 2013). Therefore, it is of particular interest to further understand factors that influence the distribution and composition of bee communities, their traits, and biotic interactions. At Mt. Kilimanjaro, bee communities have been recorded, but so far mainly morphological traits and the influence of temperature and resources as drivers of species richness and seasonal patterns have been studied (Classen et al., 2015, 2017; Mayr et al., 2020; Mayr et al., 2021). Here, we aim to deepen our understanding of so far neglected traits and interactions of bees which shape the turnover of species communities along climatic gradients. We specifically address changes of cuticular chemistry, and of interactions between bees, plants (food resources), and microorganisms, as well as potential interactions of these particular factors.

These factors are expected to undergo strong changes with increasing elevation: The composition of cuticular hydrocarbons determines desiccation resistance (Gibbs & Rajpurohit, 2010) and other qualities of the cuticula, such as interspecific and intraspecific communication (Menzel et al., 2017). Available food plant resources change in composition (Hemp, 2006), abundance, and richness (Peters et al., 2016) with increasing elevations. Associations with mutualistic bacteria may be temperature dependent in ectothermic insects. To our best knowledge, the response of these factors to elevational gradients is mostly uninvestigated in bees, taken for themselves as well in context with each other.

From previous studies, it is known that *Lasioglossum* bees show a widespread distribution along the elevational gradient at Mt. Kilimanjaro, from savannah lowlands up to the lower alpine zone and it is the only genus found throughout the whole elevational gradient (Classen et al., 2015). *Lasioglossum* is also the largest and most widely distributed bee genus on earth and is generally considered an intriguing model system known for strong inherent variations regarding ecology, behavior (Gibbs et al., 2012), and resulting adaptability to different habitats.

We are thus interested in how variation of traits and ecological interactions translates into the genus's widespread occurrence along the extensive elevational gradient at Mt. Kilimanjaro and specifically investigated the following research questions:

1. Is there a turnover of *Lasioglossum* species along the elevational gradient and do we detect cryptic species by phylogenetic inference based on DNA barcoding?

Based on this and to further assess species‐specific adaptations to the changing environment in this genus, we investigated three different traits to contrast species at different elevations:

- Do cuticular chemical profiles change along the elevational gradient, particularly with respect to saturated compounds, involved in the cuticula's function as a desiccation barrier?
- Does the composition of pollen food resources change along the elevational gradient of Mt. Kilimanjaro?
- Are these elevational changes in climate, diet, or cuticular chemistry reflected by structural changes in the composition of associated microbiomes?

## 2 MATERIAL AND METHODS

### 2.1 Study area and bee sampling

The study was conducted in the dry season between July and September in 2015 on the southeastern slopes of Mt. Kilimanjaro (Tanzania, East Africa, Figure S1), which is the highest free‐standing mountain of the world. Consequently, a lot of different habitat types are present from 700 m to 5,895 m a. s. l. and cover savannah lowlands, different mountain rainforests at mid‐elevations, and alpine vegetation at the highest elevations. The mean annual temperature decreases about ~0.56°C per 100 m, from 25°C in the lowlands to −8°C at the summit (Appelhans et al., 2016). The mean annual relative humidity is highest in the forest belt at ~2,200 m a.s.l. (Appelhans et al., 2016) and shows a hump‐shaped distribution along the elevational gradient (Figure S2). We caught halictine bees on 18 sites, equally distributed in elevation, between 830 m and 3,780 m. The collection sites were selected along mountain transect with GPS for the purpose to adequately cover different elevations and with regard to openness and flower availability in order to be able to catch the bees. The time period was chosen for relatively constant and comparable weather conditions at low and high elevations. The lower elevations were sampled around July, early after the long rainy season when the rains were not so frequent anymore, but the savannah was still green and full of flowers. The mid to higher elevations were sampled later in the dry season during August to be able to catch warmer and sunny days on the mountain when bees were active. The bees were caught when sitting on flowers or soil with sterile collection jars by hand and immediately placed on ice in an insulated flask in order to keep them below 0°C when walking down the mountain, which could be 2–3 days. At the research station, they were frozen in a freezer until they were dissected. The dissection was done under sterile conditions under a binocular microscope. The bees were dissected in head, thorax–abdomen, and gut. The thorax and abdomen were used for the analysis of the cuticular hydrocarbons. Gut and head were separately used for the analysis of the microbiome, since the surface microbiome could additionally be influenced by the composition of the cuticular chemistry. The pollen data were obtained from the gut, too. We placed the head and the gut in vials with Zymo Xpedition™ Buffer and glass beads to stabilize the bacterial DNA. We only used bees of the genus *Lasioglossum* for the analysis of pollen, microbiomes, and CHCs.

### 2.2 Chemical analysis of cuticular hydrocarbons (CHC)

CHCs were extracted with hexane, in which the thorax and abdomen were submersed for 10 min. CHC extracts were stored at 4°C and later in Germany at −20°C. Extracts were analyzed with a gas chromatograph (Agilent 7890, GC) coupled to a mass selective detector (Agilent 5975, MS). The GC (split/splitless injector in splitless mode for 1 min, injected volume: 1 µl at 300°C injector temperature) was equipped with a DB‐5 Fused Silica capillary column (30 m × 0.25 mm ID, *df* = 0.25 µm, J&W Scientific, Folsom, USA). Helium was used as carrier gas with a constant flow of 1 ml/min. The temperature program used was start temperature at 60°C, with an increase of 5°C/min until 300°C, and isotherm at 300°C for 10 min. An ionization voltage of 70 eV (source temperature: 230°C) was set for the acquisition of the mass spectra by electron ionization (EI‐MS). CHCs were classified into three main substance classes: n‐alkanes, methyl‐branched alkanes, and unsaturated compounds, with one (i.e., alkenes) or more (i.e., alkadienes, alkatrienes) double bonds. n‐Alkanes were confirmed by purchasable standards and methyl‐branched alkanes and unsaturated hydrocarbons were identified by their diagnostic ions. Retention indices were calculated and used to confirm the identification of unsaturated hydrocarbons and methyl‐branched alkanes (Carlson et al., 1998).

### 2.3 Pollen metabarcoding

Diversity analysis of pollen followed the method of Sickel et al. (2015), yet with the same extracts as for the microbial assessments: We used the combination of plant barcoding primers ITS‐S2F and ITS4R to amplify the ITS2 of the ribosomal cistron. PCR contained 5 µl 2× Phusion Master Mix (New England Biolabs, Ipswich, MA, USA), 0.33 µM each of the forward and reverse primers, 3.34 µl PCR grade water, and 1 µl DNA template. PCR conditions were as follows: initial denaturation at 95°C for 4 min, 37 cycles of denaturation at 95°C for 40 s, annealing at 49°C for 40 s, and elongation at 72°C for 40 s; followed by a final extension step at 72°C for 5 min. PCRs were performed similarly to the 16S assessment with dual‐indexed primers, in triplicates, followed by the same quantification, normalization, and final sequencing steps as described below. Also here, forward and reverse reads were joined with fastq‐join (Aronesty, 2013) and low quality (<Q20) and short (<150 bp) reads removed with USEARCH v8.0 (Edgar, 2010). Sequences were classified with USEARCH by a global search with 0.97% identity against a reference database composed from all sequences available on GenBank (Benson et al., 2013), which was created following Sickel et al. (2015).

### 2.4 Microbiome screening

The complete laboratory workflow for 16S bacterial microbiome assessment follows the strategy of Junker and Keller (2015): Stabilized DNA was isolated using the Xpedition™ Fungal/Bacterial DNA MiniPrep (also Zymo Research) following the manufacturer's instructions; however, Proteinase K was added to also facilitate the chemical disruption of pollens alongside the standard mechanical bead disruption (Bell et al., 2016; Keller et al., 2015). PCR and library preparation were performed according to a previously published dual‐indexing approach (Kozich et al., 2013). PCR was performed in triplicate for each sample in 10 µl reactions, each containing 5 µl 2× Phusion High Fidelity PCR Master Mix (New England Biolabs, Ipswich, MA, USA), 0.33 µM each of forward and reverse primer for the 16S V4 region (Eurofins MWG Operon, Huntsville, AL, USA), 3.34 µl PCR grade water and 1 µl template DNA. PCR conditions comprised an initial denaturation step at 95°C for 4 min, 35 cycles of denaturation at 95°C for 40 s, annealing at 55°C for 30 s and elongation at 72°C for 1 min, followed by final extension at 72°C for 5 min. Triplicates of a sample were combined and successful amplification was verified with an agarose gel using 5 µl. The remaining 25 µl were cleaned up and normalized between samples in DNA amounts using the SequalPrep™ Normalization Plate Kit (Invitrogen GmbH, Carlsbad, CA, USA), eluting in 20 µl. Of each sample, 5 µl normalized DNA was taken for pooling 4 × 96 samples (together with samples of other projects and laboratory control samples with the pure extraction kit) according to Kozich et al. (2013). These pools were verified for library fragment size with a Bioanalyzer High Sensitivity DNA Chip (Agilent Technologies, Santa Clara, CA, USA) and quantified with the dsDNA High Sensitivity Assay (Life Technologies GmbH, Darmstadt, Germany) and merged to a final pool. This was diluted to 2 nM and further prepared for sequencing following the Illumina Guide for DNA library preparation (lllumina Inc., 2013), obtaining a final library of 10 pM. PhiX Control Kit V3 (Illumina Inc., San Diego, CA, USA) was added as a spike‐in to ensure high‐quality reads (10%). Sequencing was performed on the Illumina MiSeq Platform (Illumina Inc., San Diego, CA, USA) using 2 × 250 bp v2 MiSeq chemistry. The cartridge of the reagent kit was additionally supplied with 3 µl each of the custom sequencing and index primers. Forward and reverse reads were joined with fastq‐join (Aronesty, 2013). Low quality (<Q20) and short (<150 bp) reads were removed, and operational taxonomic units (OTUs) clustered at 97% identity with USEARCH v8.0 (Edgar, 2010). Sequences were classified using the RDP classifier and a bootstrap threshold of 0.8. Microbiome and pollen data were imported into R (R Core Team, 2018) and managed with the *phyloseq* package (McMurdie & Holmes, 2014).

### 2.5 Phylogeny and species delineation

For phylogenetic reconstruction, the same DNA extracts were used. The cytochrome oxidase I gene (COI) was amplified with different primer combinations (forward: dgHCO (Meyer, 2003), RON (Danforth et al., 2003); reverse: dgLCO (Meyer, 2003), MAD (Danforth et al., 2003)) to successfully retrieve overlapping gene fragments merged to a consensus sequence for each specimen. These sequences were aligned using MUSCLE (Edgar, 2004), and the final alignment was 611 bp long. Supplementary reference sequences were obtained by a BLAST search against GenBank (Benson et al., 2013), with picking closely related taxa, but also others to represent all major clades of the halictine bees and three Apidae as well as three Megachilidae outgroup sequences. The tree was reconstructed using BEAST2 (Bouckaert et al., 2014) and the Yule Model (Yule, 1925) with the outgroup defined as a prior. Phylospecies were delineated using the Generalized Mixed Yule Coalescent (GMYC) method (Pons et al., 2006) as implemented in the R package *splits* (Ezard et al., 2017).

### 2.6 Climate data

The climate data for each sampling site were extracted with ArcGIS from interpolated maps of Mt. Kilimanjaro, showing the long‐term mean annual temperature and relative humidity (Appelhans et al., 2016).

### 2.7 Statistical analysis

All statistical analyses were performed in R, version 3.5.1. (R Core Team, 2018).

#### 2.7.1 CHC

We calculated the number, Shannon diversity, and evenness of CHC compounds for each sample site. Generalized additive models (gam) as implemented in the R package *mgcv* (Wood, 2011) were used to correlate the number, Shannon diversity, and evenness of CHC compounds with the elevational gradient. The data family was set to ‘gaussian’. In gam models, we set the basis dimension of the smoothing term *k* to three to avoid over‐parameterization of trend functions. We calculated the ratio between saturated and unsaturated CHCs for each sample site. Saturated compounds are n‐alkanes and methyl‐branched alkanes, and unsaturated compounds are n‐alkenes and alkadienes:

$$y=\frac{\text{n - alkanes}\left[\%\right]+\text{methyl - branched alkanes}\left[\%\right]}{\text{n - alkanes}\left[\%\right]+\text{methyl - branched alkanes}\left[\%\right]+\text{n - alkenes}\left[\%\right]+\text{dialkenes}\left[\%\right]}.$$

We further calculated the mean chain length of CHCs for each sample site:

$$y=\frac{\sum_{i=0}^{n}\text{chain length of compound}}{\text{number of different compounds}}.$$

We used linear models (lm) to calculate trends in the ratio between saturated and unsaturated CHCs and the mean chain length along the elevational gradient.

#### 2.7.2 Pollen

We transformed the abundance data to relative abundances and filtered taxa per sample with less than 1% read contribution. We calculated the number, Shannon diversity, and evenness of plant species for each sample site. Linear models (lm) and generalized additive models (gam), respectively, were used to correlate the number (lm), Shannon diversity, and evenness (gams) of plant species with the elevational gradient.

#### 2.7.3 Gut microbiota

We transformed the abundance data to relative abundances and calculated the relative number, Shannon diversity, and evenness of gut OTUs for each sample site. Linear models (lm) were used to correlate the number, Shannon diversity, and evenness of gut OTUs with the elevational gradient. Generalized additive models (gam) were used to calculate trends in abundances of Lactobacillaceae, Enterobacteriaceae, and Rickettsiaceae along the elevational gradient. The data family was set to ‘gaussian’. We checked the normal distribution of residuals with the function check_distribution of the *performance* package (Lüdecke et al., 2020). In gam models, we set the basis dimension of the smoothing term *k* to three to avoid over‐parameterization of trend functions. Trend lines derived with gams were only plotted if the significance level of the elevation term was *p* < .05.

#### 2.7.4 Comparative analyses

We analyzed the effect of temperature and relative humidity on CHCs, pollen diet, and microbiome, as well as potential effects on each other (overview about models in Table S6). First, we calculated the differences in CHC compounds or composition of pollen species and microbiome OTUs, using the metaMDS function with Bray–Curtis distances in the R package *vegan* (Oksanen et al., 2020). We applied nonmetric multidimensional scaling (NMDS) along two axes and used the axes values as explanatory and response variables in linear models. To evaluate the support for the full model and all nested models, we used the ‘dredge’ function of the *MuMIn* package (Bartón, 2018) in R. Models were ranked according to their Akaike information criterion corrected for small sample sizes (AICc) (Burnham & Anderson, 2004) and delta distances to the next best model were calculated. All best‐supported models up to ΔAICc < 3 are given in the Supplementary Information, S6. Before the analyses, all explanatory variables were standardized by z‐transformation, using the ‘scale’ function in R in order to facilitate the comparability of their effect strength.

## 3 RESULTS

### 3.1 Elevational phylogenetics of halictid bees

**FIGURE 1:**
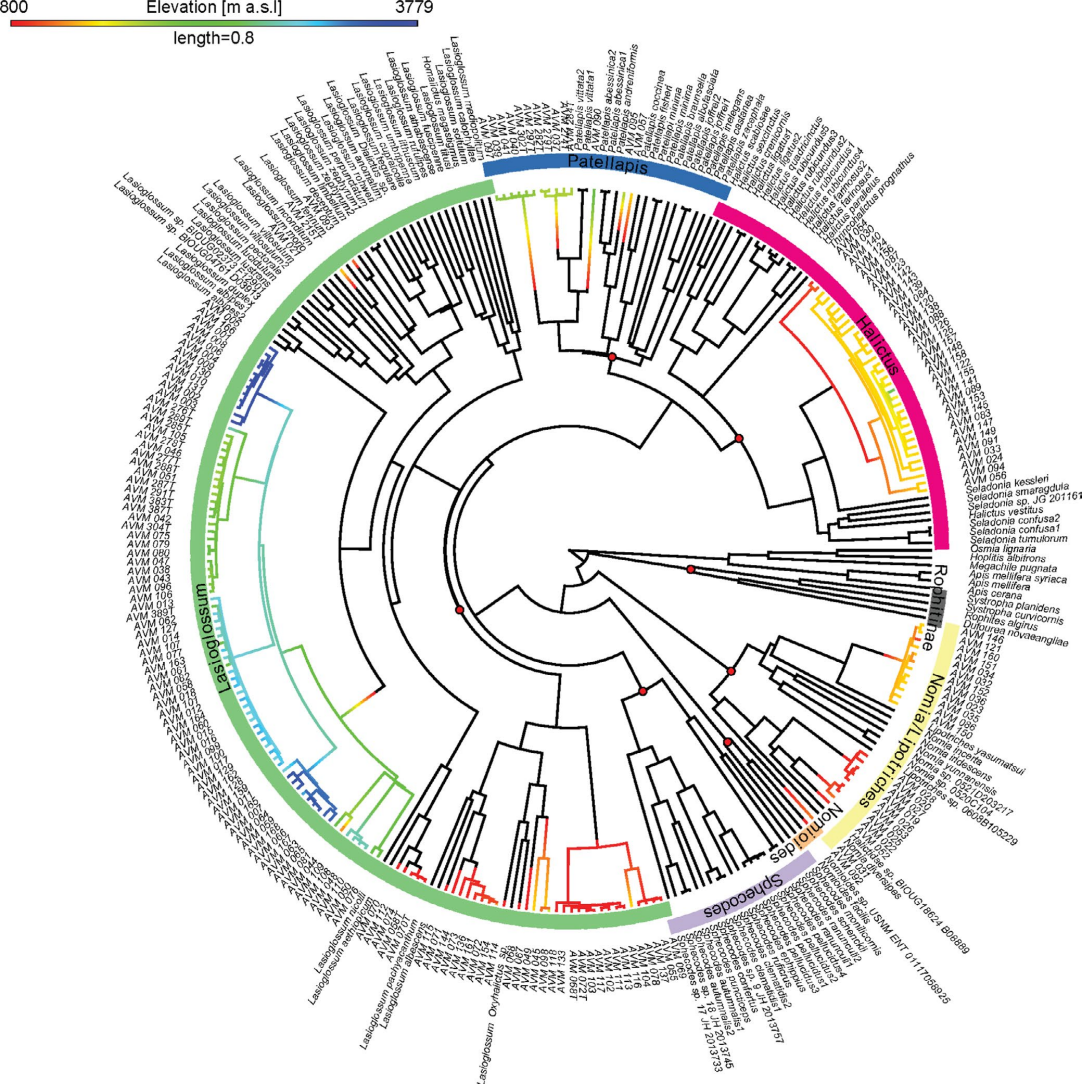
Bayesian phylogenetic tree of the collected halictid bees and reference sequences from GenBank (Benson et al., 2013) constructed on the COI gene with BEAST2, including representatives from all major clades of the Halictidae and three Apidae as well as three Megachilidae outgroup sequences. Colored branches belong to the collected halictid bees and indicate the elevation where they have been collected, black ones belong to reference sequences. Colors in the outer orbital indicate the genera and in the case of Rophitinae, the subfamily. No colors in the outer orbital belong to Apidae and Megachilidae as outgroup sequences. Node bubbles in red represent the different taxonomic groups of the Halictidae collected or used as outgroups

We caught 167 individuals of halictid bees between 830 and 3,780 m a. s. l. at the southern slopes of Mt. Kilimanjaro. The bees were not larger than 1 cm in size and the phylogenetic analysis of the COI revealed that individuals belonged to different genera: 98 *Lasioglossum,* 32 *Halictus*, eight *Patellapis* bees (Halictinae), 13 *Lipotriches,* and ten *Pseudapis* (both Nomiinae), one *Nomioides* (Nomiodinae), and five other halictid bees which could not be identified at genus level. We distinguished 26 phylospecies of *Lasioglossum* (Figure 1), eight *Halictus,* five *Patellapis,* one *Lipotriches*, and four *Pseudapis*. *Lasioglossum* was present along the whole gradient with highest diversity at lower elevations. At high elevations, our barcoding approach revealed four different *Lasioglossum* phylospecies, where previously only one morphospecies could be distinguished. The phylogenetic tree showed clear elevational patterns of species in their distribution range indicating strong phylogenetic clustering at high elevations and a more dispersed phylogenetic structure at low elevations (Figure 1). The *Lasioglossum* subgenera *Ctenonomia*, *Dialictus*, and *Oxyhalictus* occurred rather at lower elevations, while *Evylaeus* was rather found at mid to high elevations.

### 3.2 Cuticular hydrocarbon composition and diversity

**FIGURE 2:**
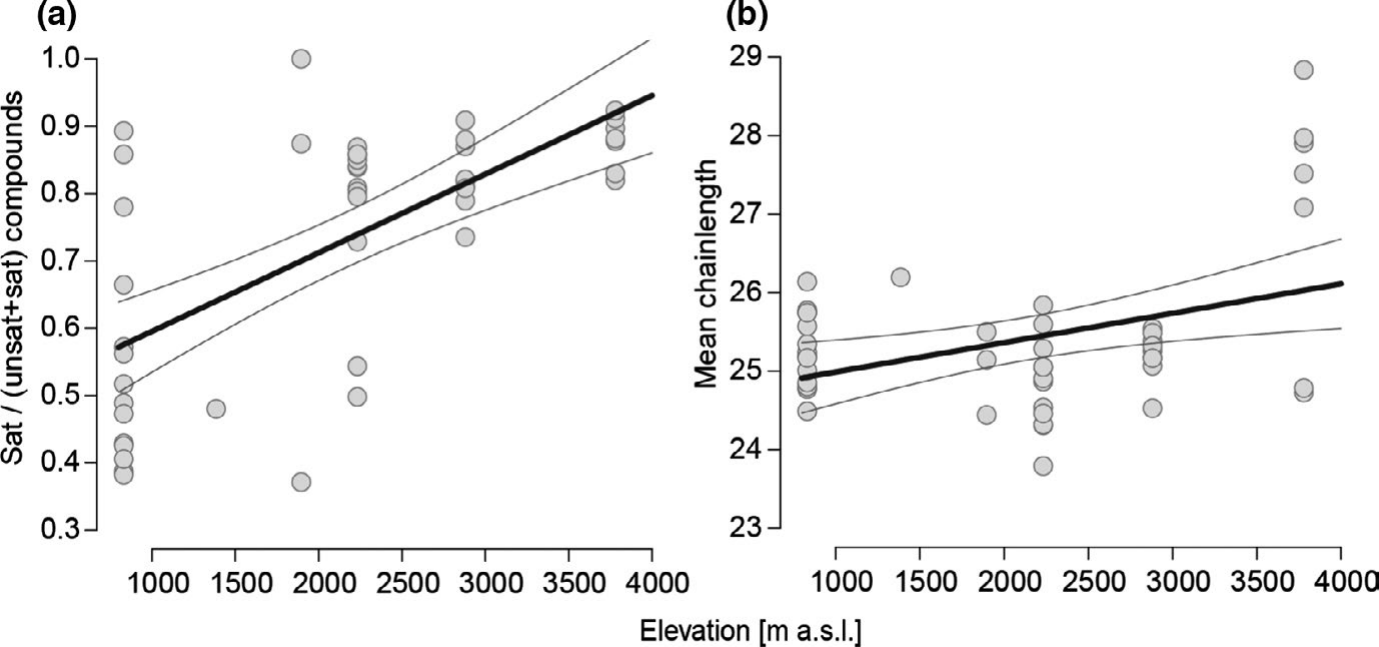
CHC of *Lasioglossum* bees along the elevational gradient. (a) Relative amounts of saturated (sat) to unsaturated and saturated (unsat + sat) CHC compounds increased with elevation (LM: *R* ^2^ = 0.42, *p* < .001). (b) The mean chain length of CHC increased with elevation in saturated compounds (LM: *R* ^2^ = 0.13, *p* < .01). Shown are trend lines from model fits with standard deviation

**FIGURE 3:**
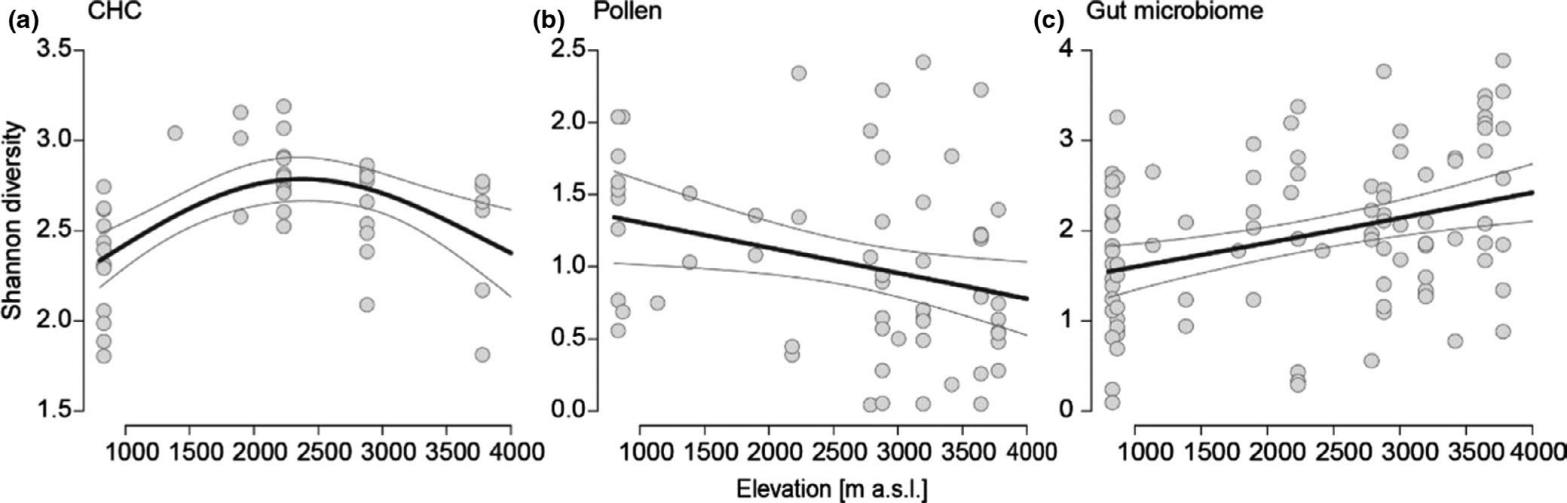
Shannon diversity of CHC compounds, pollen diet, and gut microbiome of *Lasioglossum* bees along the elevational gradient. (a) CHC compounds have the highest diversity ad mid‐elevations (GAM: ED = 34%, *p* < .001). (b) The pollen diversity decreases with elevation (LM: *R* ^2^ = 0.07, *p* = .03). (c) The diversity of the gut microbiome increases with elevation (LM: *R* ^2^ = 0.11, *p* < .001). Shown are trend lines from model fits with standard deviation

To test whether CHC profiles change along the elevational gradient, particularly in compounds which act as a desiccation barrier, we analyzed the relative amounts of saturated compounds in relation to unsaturated compounds, mean chain length of compounds, and the compound richness. The ratio of saturated to unsaturated compounds increased with elevation (*R* ^2^ = 0.42, *p* < .001, Figure 2a), as well as mean chain length in saturated (*R* ^2^ = 0.13, *p* < .01, Figure 2b), but not in unsaturated compounds (*R* ^2^ = −0.02, *p* = .56). While up to 1,500 m n‐alkanes and unsaturated hydrocarbons accounted almost equally to overall composition, n‐alkanes accounted for up to seven times the relative amount of unsaturated hydrocarbons at highest elevations. At mid‐elevations, we found a high relative abundance of methyl‐branched alkanes, which were of minor importance at lower and higher elevations (Figure S3.2). Highest Shannon diversity of CHC compounds (GAM: ED = 34%, *p* < .001, Figure 3a) as well as highest richness of CHC compounds (*R* ^2^ = 0.50, *p* < .001, Figure S3.1a) occurred at mid‐elevations.

### 3.3 Pollen diversity

To understand bee‐foraging patterns along the elevational gradient, we studied the richness and composition of pollen in bee guts using plant ITS2 metabarcoding. We obtained 615,552 sequences after merging and quality filtering. Individual samples yielded a mean of 10,433 sequences and 28 samples with less than 100 reads were removed to avoid throughput‐related underestimations in diversity. We detected in total 170 plant species from which pollen samples originated with on average 15 species per sample (range 3–26). Dominant plant families were Asteraceae, Ericaceae, and Fabacaeae (Figure S4.2). Shannon diversity of collected pollen decreased along the elevational gradient (*R* ^2^ = 0.07, *p* = .03, Figure 3c) as well as evenness (*R* ^2^ = 0.09, *p* = .03, Figure S4.1b), but not pollen richness (*R* ^2^ = −0.01, *p* = .46, Figure S4.1a). Asteraceae were the dominant pollen resource for bees along the whole elevational gradient, but especially at highest elevations, where they contributed up to 100% of the consumed pollen (Figure S4.2 and Figure S4.3).

### 3.4 Microbial communities

**FIGURE 4:**
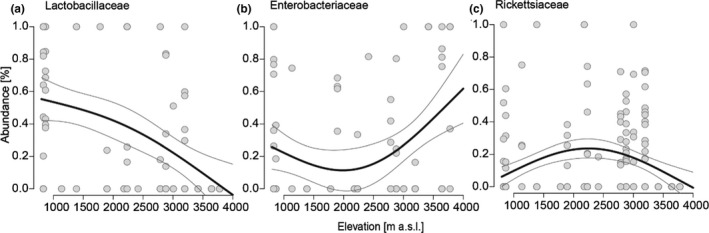
Major bacterial families in the gut microbiome of *Lasioglossum* bees along the elevational gradient. (a) The relative abundance of Lactobacillaceae decreases with elevation (GAM: ED = 21.5%, *p* < .001). (b) The relative abundance of Enterobacteriaceae increases parabolic with elevation (GAM: ED = 12%, *p* < .01). (c) The relative abundance of Rickettsiaceae shows highest relative abundances at mid‐elevations (GAM: ED = 21.5%, *p* < .001). Generalized additive models were used to estimate trends of elevational richness (Gaussian family, basis dimension (*k*) = 3). Shown are trend lines from model fits with standard deviation

To investigate whether changing environmental conditions, cuticular chemistry, diet, and species identity along the elevational gradient at Mt. Kilimanjaro lead to a turnover in microbiome composition, we used 16S DNA metabarcoding to analyze gut and surface microbiomes. In total, we obtained 10,092,494 sequences after merging, quality filtering as well as mitochondria and chloroplast sequence removal. Individual samples had on average 26,093 reads, and samples with less than 1,000 reads were removed from the following analyses to avoid underestimations in diversity. Shannon diversity of OTUs in the gut microbiome increased with elevation (*R* ^2^ = 0.11, *p* < .001, Figure 3c), as well as OTU richness and evenness (richness: *R* ^2^ = 0.12, *p* < .001; evenness: *R* ^2^ = 0.09, *p* < .01, Figure S5.1a,b). The community composition also varied along the elevational gradient with Lactobacillaceae, Enterobacteriaceae, and Rickettsiaceae as the three dominant groups (Figure 4, Figure S5.2a). While Lactobacillaceae declined with elevation (ED = 21.5%, *p* < .001, Figure 4a), and Rickettsiaceae showed highest relative abundances at intermediate elevations (ED = 21.5%, *p* < .001, Figure 4c), the percentage of Enterobacteriaceae increased with elevation, mainly due to an increase in the genus *Sodalis* (ED = 12%, *p* < .01, Figure 4b). Surface microbiomes showed similar patterns, yet with general higher prevalence of Comamonadeaceae, Moraxellaceae, Oxalobacteraceae, Pseudomonaceae, and Xanthomonadaceae (Figure S5.2b).

### 3.5 Interactive effects of environment, phylospecies, pollen resources, microbiomes, and cuticular hydrocarbon profiles

**FIGURE 5:**
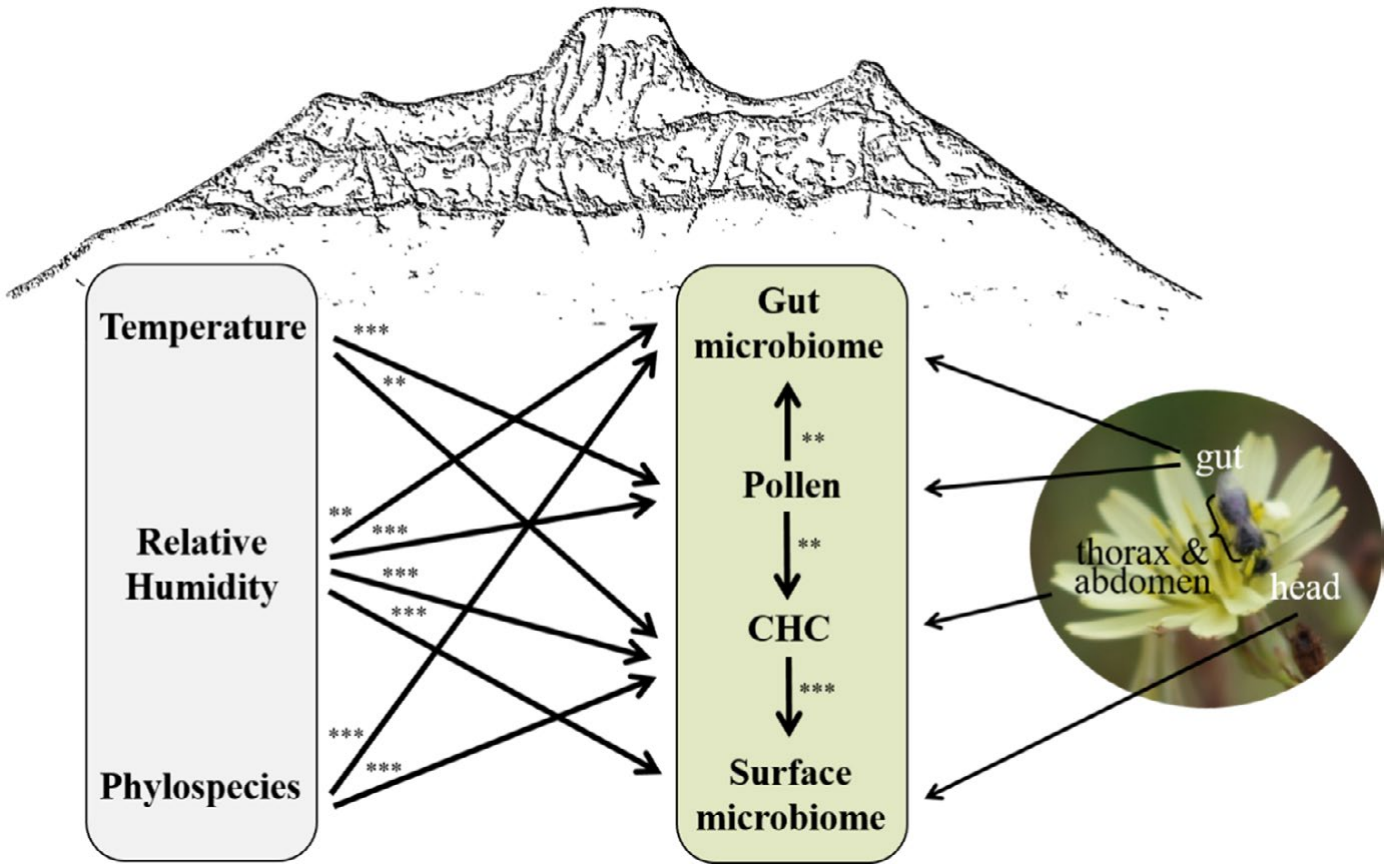
Schematic analysis and significance of drivers of gut microbiome, pollen diet, CHC profile, and surface microbiome of *Lasioglossum* bees. Shown are significant drivers, selected from best models, based on AICc values. Different body parts were used to obtain CHC, pollen, and microbiome data. To calculate models with compositional data, we calculated Bray‐Curtis distances and applied nonmetric multidimensional scaling (NMDS) along two axes and used the axes as explanatory and response variables, respectively. Temperature and relative humidity, as well as the phylospecies identity, were included as explanatory variables in linear models as well. Further details and results on the models are displayed in Table S7

Our results reveal that multiple traits and interactions of *Lasioglossum* bees changed along the elevational gradient. In the next step, we analyzed how they were shaped by environmental conditions (temperature, relative humidity) and bee phylogeny and how traits and biotic interactions were linked. Best models, based on AICc selection, revealed that climate, CHC composition, pollen composition, gut, and surface microbiomes are correlated with relative humidity, while pollen and CHC composition additionally responded to increasing temperature. Changes in pollen and gut microbiome composition correlated with each other, but also pollen and CHC, as well as CHC and surface microbiome composition were correlated. Additionally, gut microbiomes and the CHCs were mostly specific for individual bee species (Figure 5, Table S6).

## 4 DISCUSSION

In this study, we revealed cryptic species with elevation‐dependent phylogenetic relationships along Mt. Kilimanjaro, hidden biotic interactions, and chemical adaptations to changing environmental parameters. Above 3,000 m a.s.l., more *Lasioglossum* species were identified with DNA barcoding than previously morphologically identified (Classen et al., 2015). The strong elevational zonation of species implies niche formation and clustering of species with elevation‐dependent adaptations. The widespread of *Lasioglossum* along the whole gradient supports the great evolutionary adaptability known for the genus and the consequential distribution throughout many types of habitats (Gibbs et al., 2012).

### 4.1 Elevational changes shape cuticular hydrocarbon composition and diversity

In line with the cuticula's function as a desiccation barrier, we found higher relative amounts of saturated compared with unsaturated CHC compounds, and increased chain lengths in saturated compounds with higher elevations. Higher relative amounts of unsaturated compounds (n‐alkenes) in a CHC profile lead to more water permeability than higher relative amounts of saturated CHCs (n‐alkanes and methyl‐branched alkanes) (Gibbs & Pomonis, 1995). However, waterproofing can be improved not only with higher portions of saturated CHC, but also by longer CHC chain lengths (Menzel et al., 2017). Mean annual relative humidity is lowest at the base of the mountain, peaks at about 2,500 m a. s. l. and slightly decreases towards the summit at Mt. Kilimanjaro (Figure S2), and the correlation between CHC composition and relative humidity confirms our hypothesis that the CHC profiles of the investigated bees are adapted to the abiotic environment. We also found CHC composition to be phylospecies‐specific, as also shown in other bees (Pokorny et al., 2014), which implies that *Lasioglossum* species are niched along the elevational gradient according to their cuticular desiccation resistance. An increase in desiccation resistance has also been found for *Drosophila* species at high elevations with very low relative humidity (Parkash et al., 2008). In our study, region relative humidity is, however, also low in savannah habitats at low elevations, and it is unclear why relative amounts of saturated CHC do not account for this. Possible explanations might be that bees at high elevations have to cope with lower relative amount of oxygen, thus keep the tracheae more open, and consequently lose more water by breathing (Chown & Gaston, 1999). Secondly, when bees loose water, they also cool down through evaporation enthalpy, which might be critical in environments with low ambient temperatures. We also found a correlation between temperature and CHC composition as shown by Michelutti et al. (2018). Therefore, probably temperature and relative humidity are both important drivers with regard to water loss. This might be reflected as well in behavioral adjustments, which bees might be able to afford at low, but not high elevations. At low elevations, with generally high temperatures, bees can easily hide in microhabitats rather than forage if the weather conditions are too dry for flying and have a potentially larger foraging window. However, at higher elevations, the bees must use every opportunity to uptake energy as soon as temperatures are high enough to fly (Classen et al., 2015), which coincides with low relative humidity.

### 4.2 Dominance of Asteraceae pollen in the diet of *Lasioglossum*

The turnover of the plant community along the elevational gradient (Hemp, 2006) was reflected in the bee's diet. We found different plant families with different abundances in the diet, as well as a decrease in the Shannon diversity along the elevational gradient. Asteraceae pollen dominated the *Lasioglossum* food throughout the entire gradient, even though there was no significant trend with elevation. Nevertheless, in some samples the Asteraceae pollen made up to 100% of the diet at highest elevations. This reflects the dominance of Asteraceae upward from the forest and *Erica* belt and the ability of Asteraceae to grow in open and dry habitats (Judd et al., 2016). Even though the Asteraceae offer an abundant pollen source (Goulson et al., 2005), they are avoided by most polylectic bee species or just visited for nectar (Müller & Kuhlmann, 2008). Possible explanations are that pollen of Asteraceae lacks essential nutrients and is hard to degrade (Praz et al., 2008), has very low protein content (Roulston et al., 2000), and often contains toxic compounds (Nicolson & Human, 2013). Bees foraging on such resources need special physiological adaptations, for example, detoxification abilities (Praz et al., 2008). The *Lasioglossum* species found along the whole gradient seem to be able to manage the mentioned particularities of Asteraceae pollen, which could be an advantage over other bees that are found only at lower regions. Pure Asteraceae diet has however also disadvantages for Asteraceae specialists, such as stunted growth (Williams, 2003) and delayed development (Praz et al., 2008; Williams, 2003). Pollen mixing and dilution is a strategy for bees to cope with unsuitable pollen diet (Eckhardt et al., 2014), a strategy, which we observed across all samples, throughout the entire elevational gradient, even though to a lesser degree at highest elevations.

The benefits for plants are not far to seek: By chemically protecting their pollen, they avoid that unspecific pollinators collect pollen and thereby maximize pollen transfer to conspecifics (Müller & Kuhlmann, 2008). However, also bees foraging on Asteraceae pollen have an advantage through their diet: Asteraceae pollen usually has antimicrobial properties (Müller & Kuhlmann, 2008) and Asteraceae specialists are known to be less often parasitized than generalists (Spear et al., 2016). Beyond *Lasioglossum* however, other specialized wild bees visit Asteraceae (Müller et al., 2006). At Mt. Kilimanjaro for instance, we found Asteraceae in the diet of other halictids such as *Halictus* and *Patellapis*, which we did not detect though at higher elevations (data not shown in this manuscript). Concluding, the restricted resource diversity likely contributes to the limited bee diversity found at higher elevations, yet is not the sole explanation.

### 4.3 Drivers of microbiotic associations in *Lasioglossum* bees

We observed strong changes in the composition of bacterial microbiomes, with a general trend of diversity and evenness increasing with elevation. So far for bees, only honey bee microbiomes have been investigated along elevational gradients, whereby the microbial community changed between lower and higher elevations along a gradient of 2,270 m (Sudhagar et al., 2017). However, honey bees as highly eusocial insects are to a certain degree capable to compensate several factors changing with the gradient, foremost temperature, and temperature‐mediated food acquisition. Thus, they have greater control over shaping microbial conditions suitable for their associates. By contrast, wild bee microbiomes are generally assumed to be more influenced by environmental factors (Keller et al., 2013; Rothman et al., 2018; Voulgari‐Kokota et al., 2018), and the transitions of these factors along an elevational gradient might as well be of stronger fitness relevance than for honey bees. At Mt. Kilimanjaro, we found that some bacterial families became more prominent at higher elevations, while others decreased.

Especially, the relative microbiome contribution of Lactobacillaceae strongly declined with increasing elevation. The family is well known to include important bee symbionts, which fulfill functions in nutrient allocation and pollen digestion (Vásquez & Olofsson, 2009), as well as for the immune system (Servin, 2004; Vásquez et al., 2012). For honey, bumble, and stingless bees, as well as for the solitary genera *Osmia* and *Megachile*, previous studies place Lactobacillaceae among the most abundant bacteria (McFrederick et al., 2017; Voulgari‐Kokota et al., 2018). This was also true in our study, but only at lower elevations. Lactobacillaceae are mostly moderately heat‐tolerant, but show inhibited growth at lower temperatures, with few specialized exceptions (Matejčeková et al., 2016; Niemand & Holzapfel, 1984). With a decrease in their contribution at higher elevations, we suggest that relevant strains are unable to maintain growth under extended cold conditions. They are thus reduced as associates at higher elevations and the functional consequences on nutrition and the immune system remain unknown. Nevertheless, if pathogens also decrease along the environmental gradient, the incremental loss of Lactobacillaceae at high elevations might not be as consequential for the host as at lower elevations. We also found that Enterobacteriaceae slightly increased with elevation, but with lowest relative abundances at mid‐elevations. They include also important insect symbionts, but are more known to occur on flowers (Junker & Keller, 2015) and bee‐collected pollen (Voulgari‐Kokota et al., 2018). High temperatures appear to be more limiting for their growth than lower temperatures (Ron, 1975; Verbarg et al., 2008), which fits to the higher relative abundances at higher elevations. Rickettsiaceae showed highest relative abundances at mid‐elevations. The most prominent taxon we found within this family, *Wolbachia,* is a well‐known genus associated with insects, with influences on host behavior or manipulations towards favorable host sex ratios for the bacterium (Zeh et al., 2005). Temperature‐dependent growth is also known for this group, reducing densities at very high and low temperatures (Bordenstein & Bordenstein, 2011; Jia et al., 2009). This is consistent with the appearance of the group at mid‐elevations. Concluding, our data suggest that elevation (and most likely ambient temperature as the direct driver) is a major contributor in structuring *Lasioglossum* microbiomes, with unknown consequences for symbiotic and antagonistic interactions.

### 4.4 Potential joint effects on sociality and correlations of interactions

Because in nature many factors are connected, we also tested for interdependencies of (a) pollen diet and gut microbiome, (b) pollen diet and CHCs, and (c) CHCs and surface microbiome. We found correlations between the pollen diet and both, CHCs and gut microbiome. The influence of the pollen diet on CHCs has been already shown (Liang & Silverman, 2000; Otte et al., 2015). As well, it is known for many insects that the food influences the gut microbiome (Engel & Moran, 2013). We also found a correlation between CHCs and the surface microbiome. The correlation is plausible, because CHCs offer favorable and unfavorable substances for the growth of bacteria (Schaeffer et al., 1979). Additionally, CHCs display the first barrier to microbes and some CHCs seem to be more difficult for bacteria to assimilate, even inhibiting their growth (Pedrini et al., 2013). However, so far very little is known about the quality and quantity of CHC interactions with bacteria. If, however, factors such as diet and CHCs are more related to microbiota as previously thought, their influences, for example, on social behavior, could be much more complex than so far assumed.

Halictids show a diverse spectrum of social behavior. Some species change between solitary and social lifestyle depending on time of the year, geographic location, elevation, and other unknown factors (Michener, 2007). Unfortunately, African halictine bees poorly known with regard to their sociality (Danforth et al., 2008). CHCs are used for the communication in social insects, predominantly n‐alkenes and methyl‐branched alkanes. These compounds either decreased with elevation or mostly occurred at mid‐elevations. The prioritization of a strong desiccation barrier at higher elevations with a high amount of saturated instead of unsaturated and methyl‐branched alkanes might therefore also interfere with or even preclude social behavior. Because likewise to parasitism, the complexity of chemical profiles for communication is in a trade‐off situation with its function for desiccation prevention, as a larger amount of saturated CHC compounds reduces the viscosity of the CHC layer and reduces the compounds which are used to encode information (Howard & Blomquist, 2005; Leonhardt et al., 2016). Therefore, the results of the cuticular analysis indicate that *Lasioglossum* might be solitary at higher elevations (Eickwort et al., 1996) and social at lower elevations at Mt. Kilimanjaro.

Additional support comes from the microbiome assessment. *Sodalis* was the most dominant Enterobacteriaceae genus in our study and in a previous study identified as the most important group for distinguishing between solitary and social behavior in halictid bee (Rubin et al., 2018). At lower elevations, we found a higher diversity of *Sodalis* OTUs, which might reflect changes in social behavior in our system as well. A third restriction of sociality might come with the low protein content of Asteraceae (Praz et al., 2008), which is the main pollen source at high elevations. Low protein pollen leads to prolonged larval developmental times (Praz et al., 2008). However, sociality is strongly linked to season length, that is, the season needs to be long enough to permit two annual broods (Field et al., 2010). The poor pollen quality could therefore prevent more than one annual brood. The investigated traits thus suggest that sociality is unlikely at higher elevations at Mt. Kilimanjaro. Nevertheless, different subgenera of *Lasioglossum* bees, such as *Ctenonomia*, *Dialictus*, *Evylaeus*, and *Oxyhalictus* seem to inhabit different elevations which are known to potentially occur in solitary and social forms (Danforth et al., 2008). Therefore, to prove the hypothesis that CHCs, the microbiome, and/or the pollen diet restrict or favor social behavior, it would be necessary though to search for nests and queens and clarify, whether a change in sociality occurs along the gradient.

## 5 CONCLUSION

Insects have to adapt to physiological and biotic constraints in order to occur in habitats with different climatic conditions, such as along elevational gradients. This requires multiple adaptations in (life‐history) traits and biotic interactions. Our findings reveal the multiple facets of *Lasioglossum* bee ecology along an elevational gradient at Mt. Kilimanjaro in Tanzania. We found strong changes in the surface profiles of the epicuticle, in collected food resources, as well as in associated bacterial communities, which seem to be functionally connected. Our approach with the intensive use of molecular tools help us to understand not only the direct, physical impact of elevation on bees, but also inherent physiological and phylogenetic constraints as well as changes in interactions with food resources and microbiota. The interplay among these factors directs to exciting new hypotheses and underpins the relevance of investigating biotic interactions on multiple levels to better understand species distributions and potential consequences of global climate change.

## CONFLICT OF INTEREST

The authors declare no competing interests.

## AUTHOR CONTRIBUTIONS

**Antonia V. Mayr:** Conceptualization (lead); data curation (lead); formal analysis (lead); investigation (lead); methodology (equal); resources (lead); visualization (lead); writing‐original draft (lead); writing‐review & editing (lead). **Alexander Keller:** Conceptualization (lead); formal analysis (lead); funding acquisition (lead); methodology (lead); resources (lead); software (lead); supervision (lead); visualization (equal); writing‐review & editing (equal). **Marcell K. Peters:** Conceptualization (equal); formal analysis (equal); supervision (equal); writing‐review & editing (equal). **Gudrun Grimmer:** Data curation (lead); methodology (lead); writing‐review & editing (supporting). **Beate Krischke:** Data curation (lead); methodology (lead); writing‐review & editing (supporting). **Mareen Geyer:** Data curation (lead); methodology (supporting); writing‐review & editing (supporting). **Thomas Schmitt:** Conceptualization (lead); formal analysis (equal); funding acquisition (equal); methodology (lead); supervision (equal); visualization (equal); writing‐review & editing (equal). **Ingolf Steffan‐Dewenter:** Conceptualization (equal); formal analysis (supporting); funding acquisition (lead); resources (lead); supervision (equal); writing‐review & editing (equal).

## Supporting information

Supplementary Material

## Data Availability

Raw sequencing data are supplied under the URL http://www.ncbi.nlm.nih.gov/bioproject/719282 with the BioProject ID PRJNA719282. The processed data will become publicly available via GFBio (https://www.gfbio.org/) according to the Rules of Procedure of the German Science Foundation (DFG). The R code can be provided upon request. The phylogenetic data is available via http://doi.org/10.5281/zenodo.4686366.
