# Supplementary material for: Cryptic species and hidden ecological interactions of halictine bees along an elevational gradient
Source: Ecol Evol. 2021 May 17;11(12):7700–12. doi: 10.1002/ece3.7605 (PMC8216903; doi:10.1002/ece3.7605)
Supplement: Supplementary file 1 — Supplementary Material [file ECE3-11-7700-s001.docx]

# Supporting Information

###### Study design

| 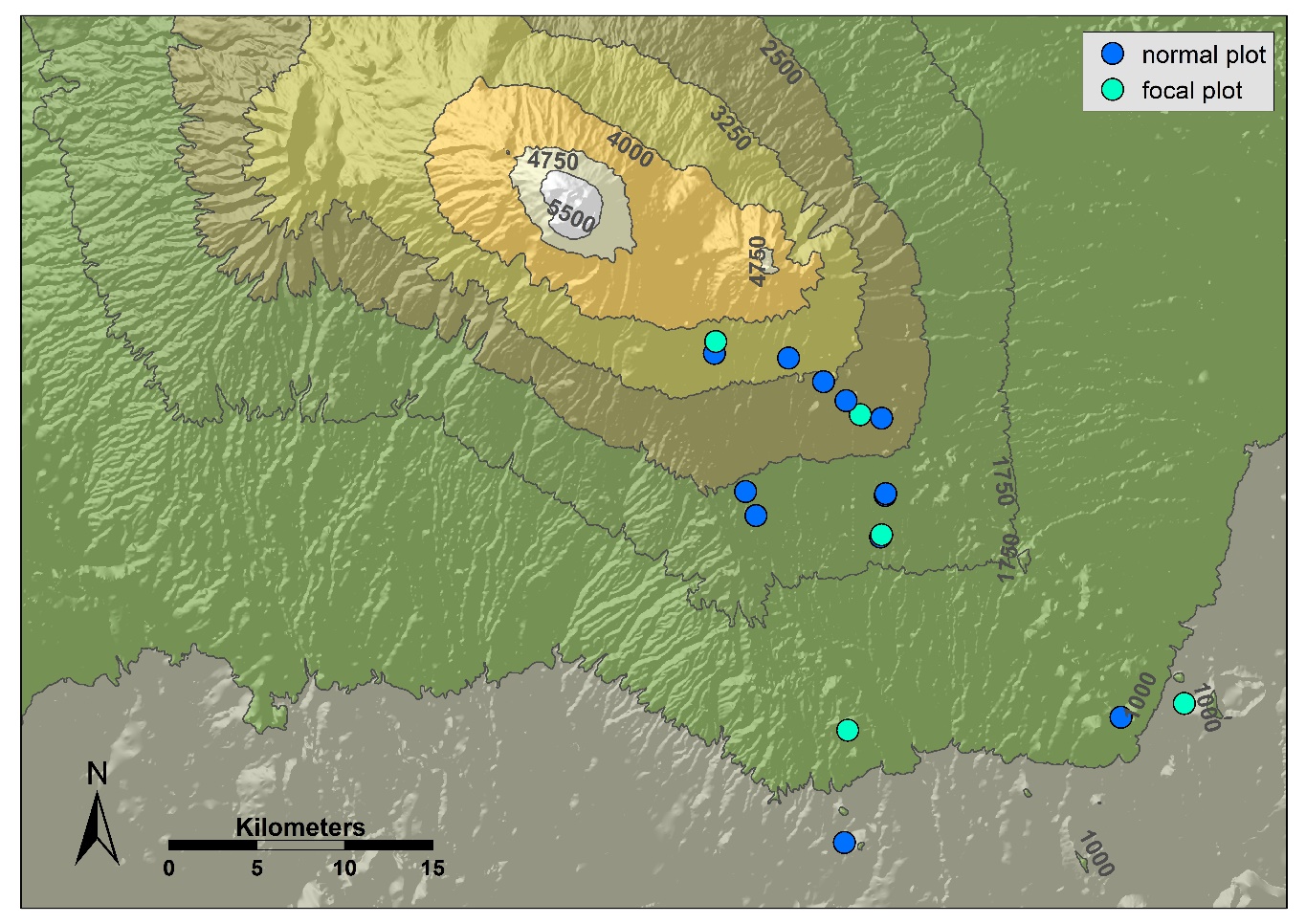 |
| --- |
| Fig. S1: Study area at Mount Kilimanjaro, Tanzania. Shown are sampling sites of *Lasioglossum* bees. Pollen and microbiota od bees were investigated on all plots, additionally CHC only on focal plots. |

###### Elevational patterns of temperature and relative humidity

| 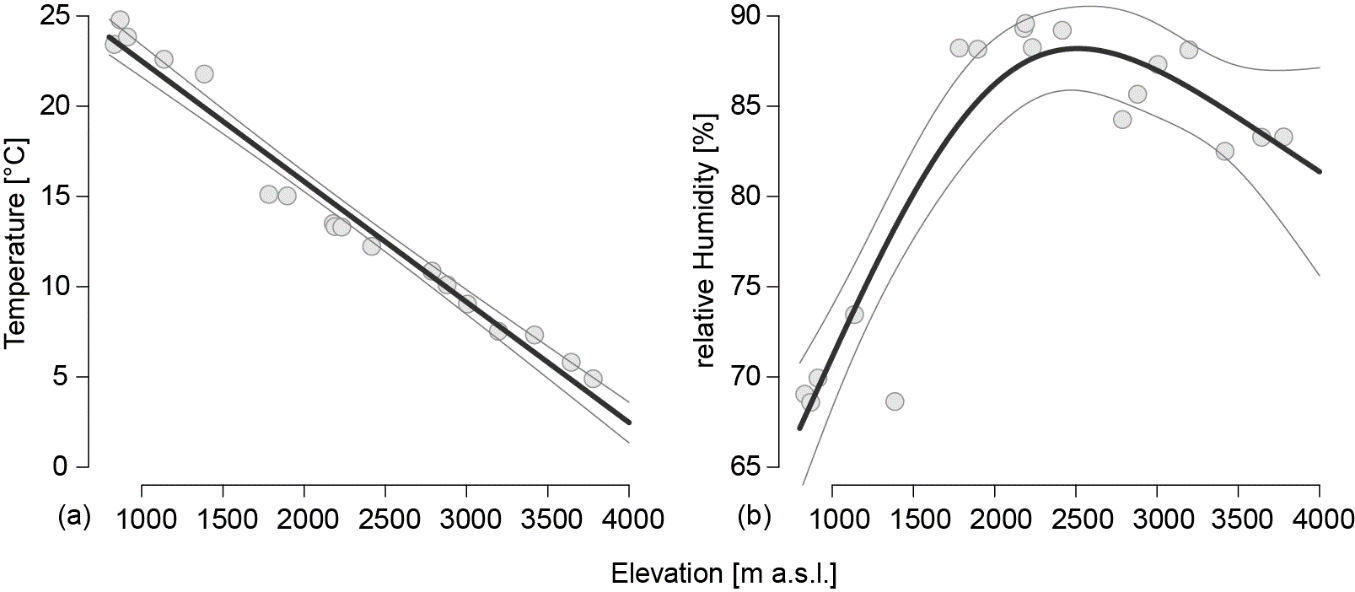 |
| --- |
| Fig. S2: Patterns of the mean annual temperature and mean relative humidity along the elevational gradient at Mt. Kilimanjaro. Linear and generalized additive models, respectively, were used to display elevational patterns of temperature and relative humidity (Gaussian family, basis dimension (*k*) = 4). (a) Temperature declines with elevation (R² = 0-97, p < 0.001), while (b) relative humidity shows a humped-shaped distribution (ED = 87, p < 0.001). Shown are trend lines from model fits with standard deviation. |

###### Elevational diversity and composition of CHC

| 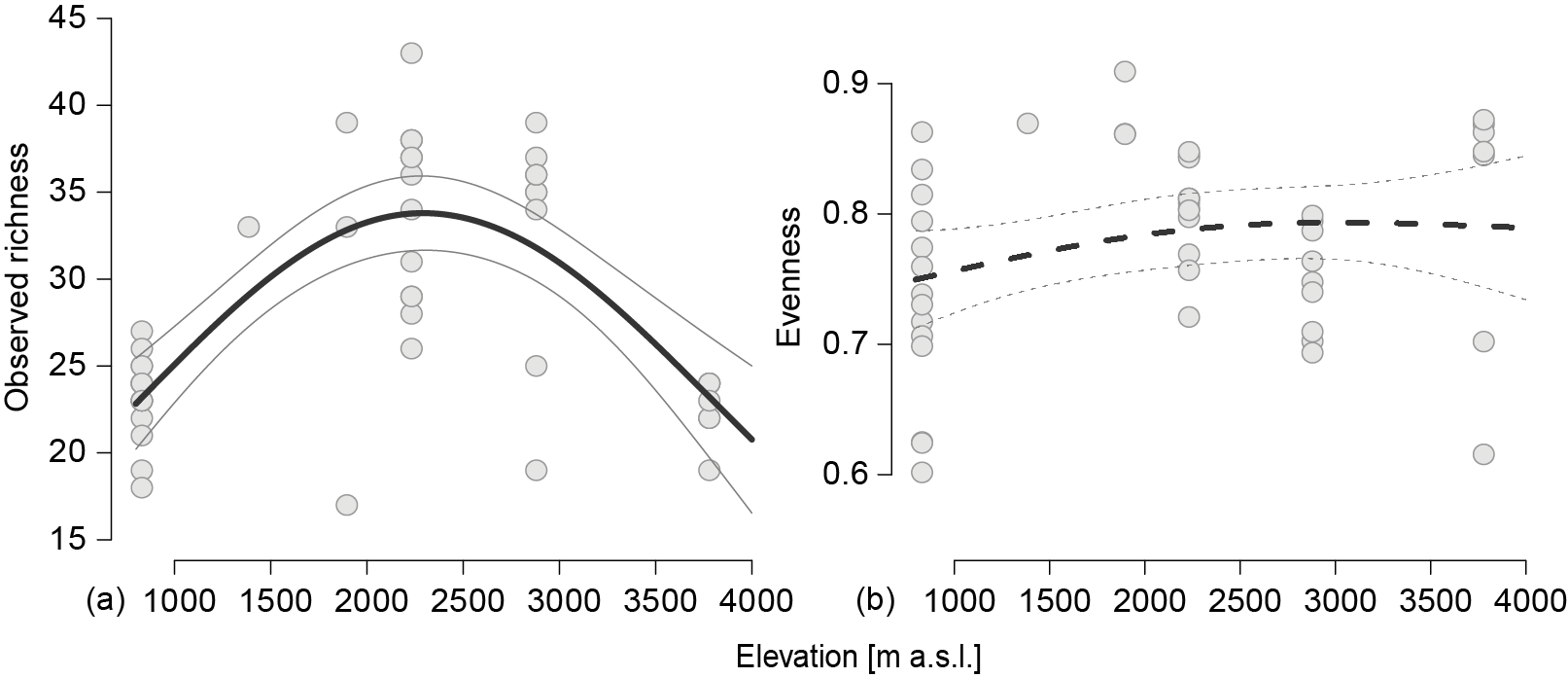 |
| --- |
| Fig. S3.1: Alpha diversity measures of CHC of *Lasioglossum* bees along the elevational gradient of Mt. Kilimanjaro. Generalized additive models, were used to display elevational patterns of observed compound richness and evenness (Gaussian family, basis dimension (*k*) = 3). (a) observed compound richness shows a humped-shaped patterns with elevation (ED = 52, p < 0.001), while (b) evenness of compounds does not increase with elevations (ED = 8, p = 0.21). Shown are trend lines from model fits with standard deviation. |

| 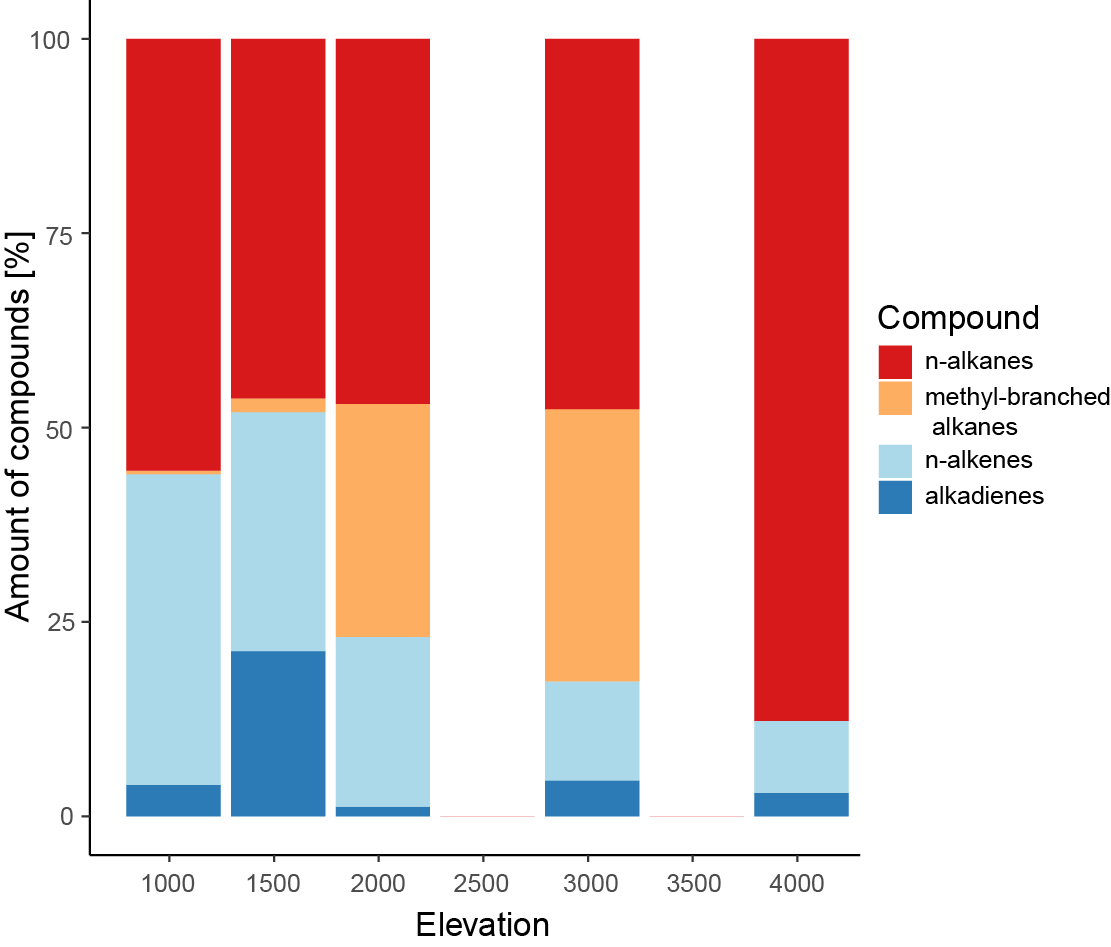 |
| --- |
| Fig. S3.2: The relative amount of CHC compound classes of *Lasioglossum* bees varies along the elevational gradient with highest proportions of n-akanes at highest elevations and highest proportions of methyl-branched alkanes at mid-elevations. |

###### Elevational richness, abundance pattern and composition of pollen

| 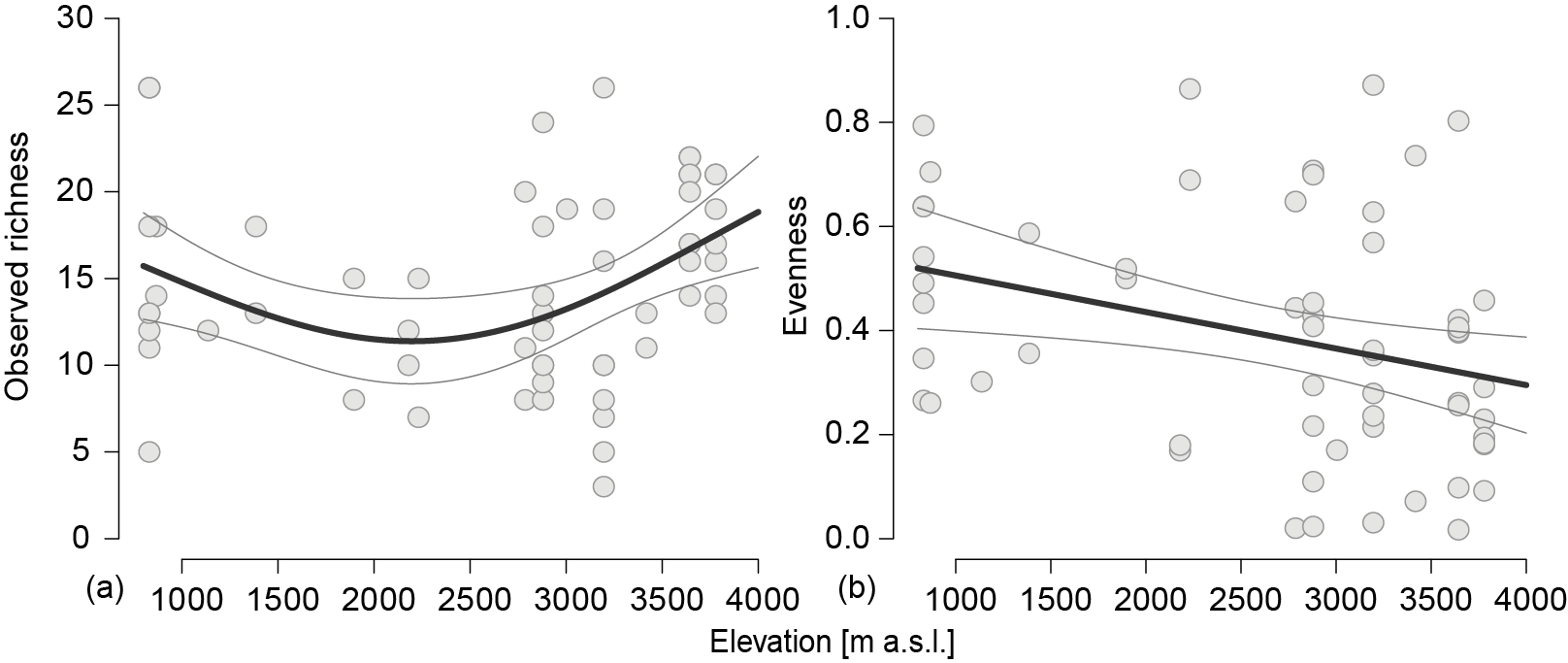 |
| --- |
| Fig. S4.1: Alpha diversity measures of pollen diet of *Lasioglossum* bees along the elevational gradient of Mt. Kilimanjaro. Linear and generalized additive models, respectively, were used to display elevational patterns of observed species richness and evenness of pollen diet (Gaussian family, basis dimension (*k*) = 3). (a) observed species richness decreases towards mid-elevations and then increases towards higher elevations (ED = 15, p = 0.02), while (b) evenness of pollen diet decreases with elevation (R² = 0.08, p = 0.02). Shown are trend lines from model fits with standard deviation. |

| 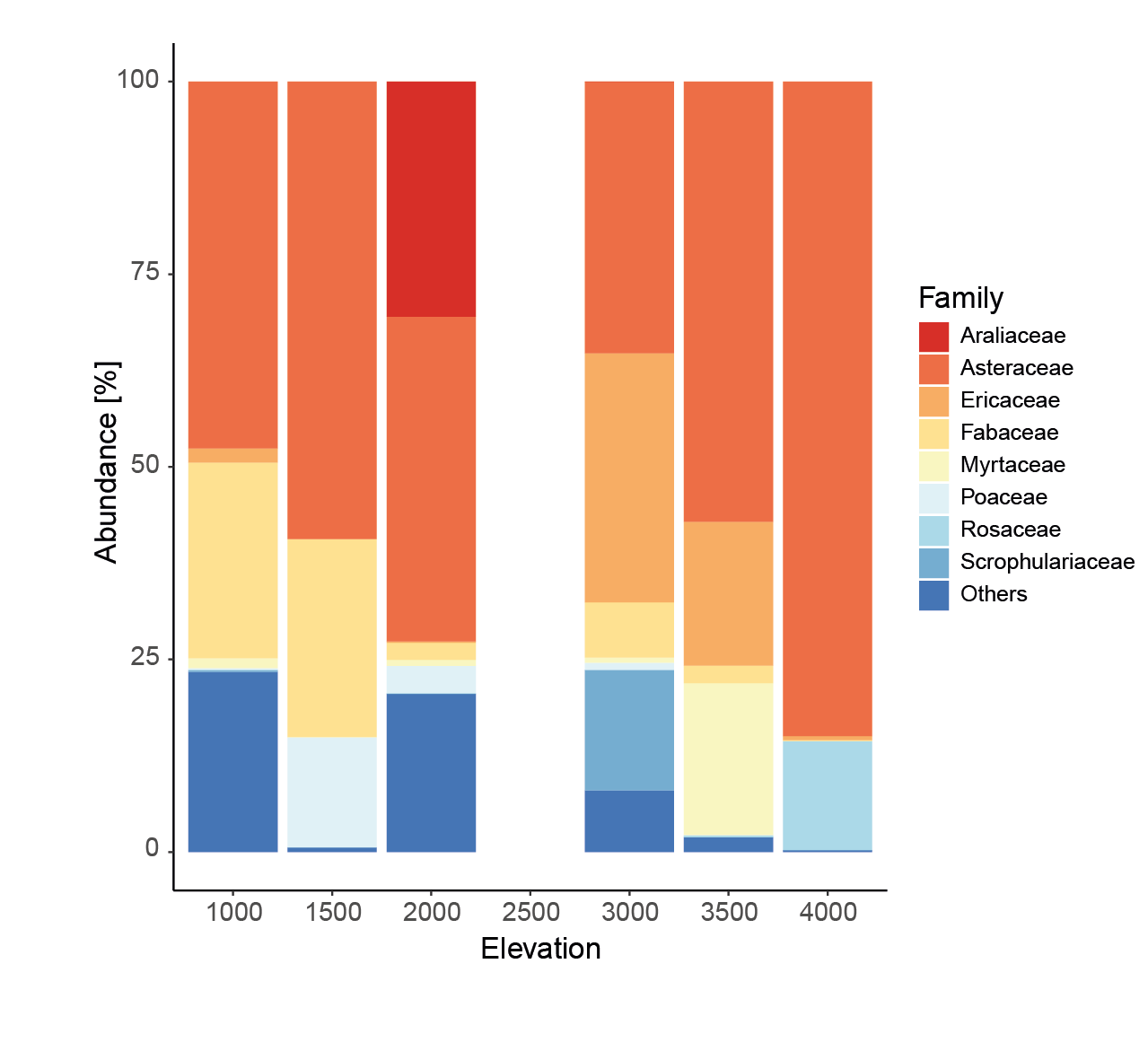 |
| --- |
| Fig. S4.2: The relative amount of plant families collected by *Lasioglossum* bees varies along the elevational gradient. The pollen diet generally comprises a high proportion of Asteraceae, with highest proportions of Asteraceae at highest elevations. |

| 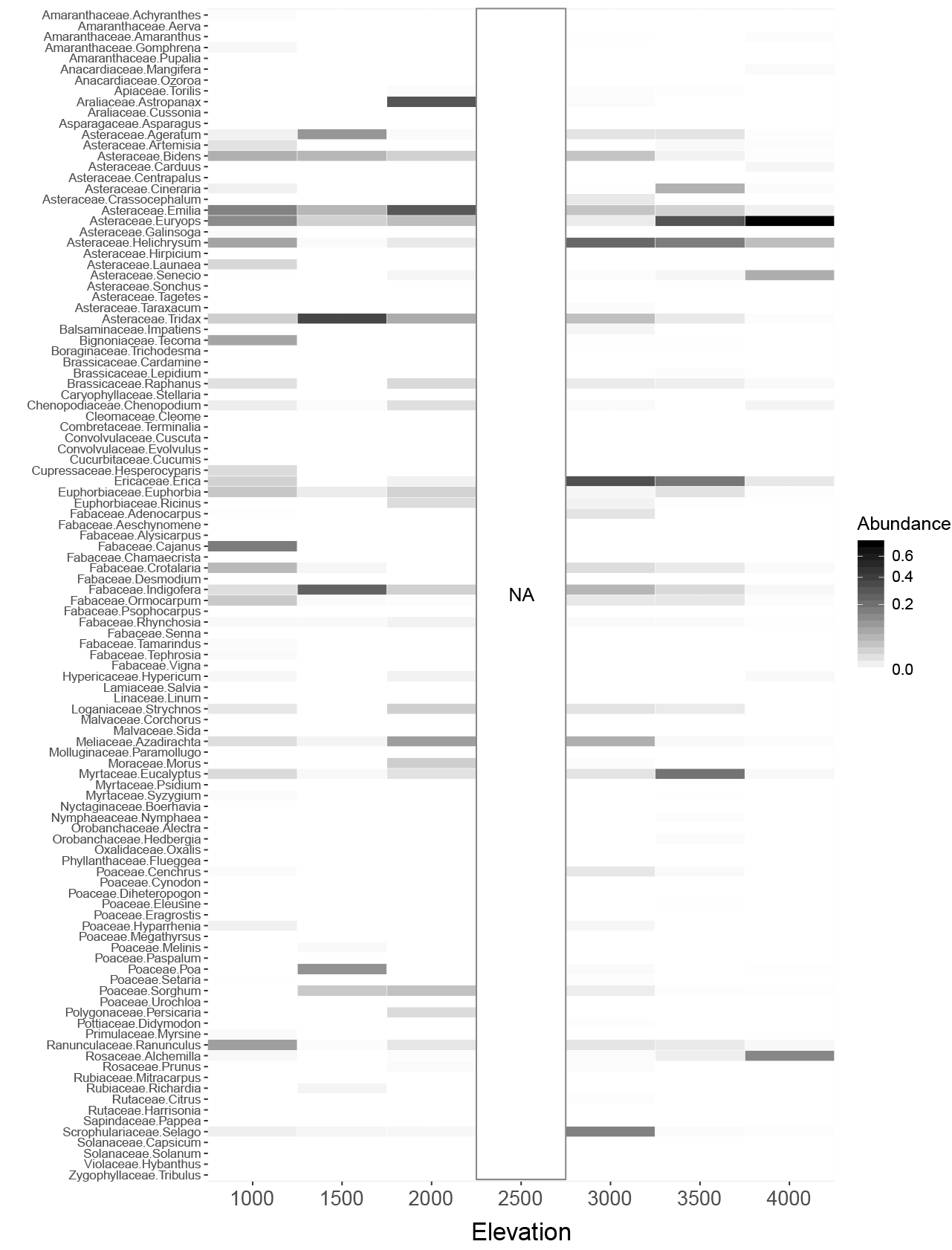 |
| --- |
| Fig. S4.3: Plant families and genera in the pollen diet of *Lasioglossum* along the elevational gradient. Shown are relative abundances of genera for each elevation. At 2500 m a.s.l. no data was available. |

###### Elevational richness pattern of gut microbiota and composition of surface microbiome

| 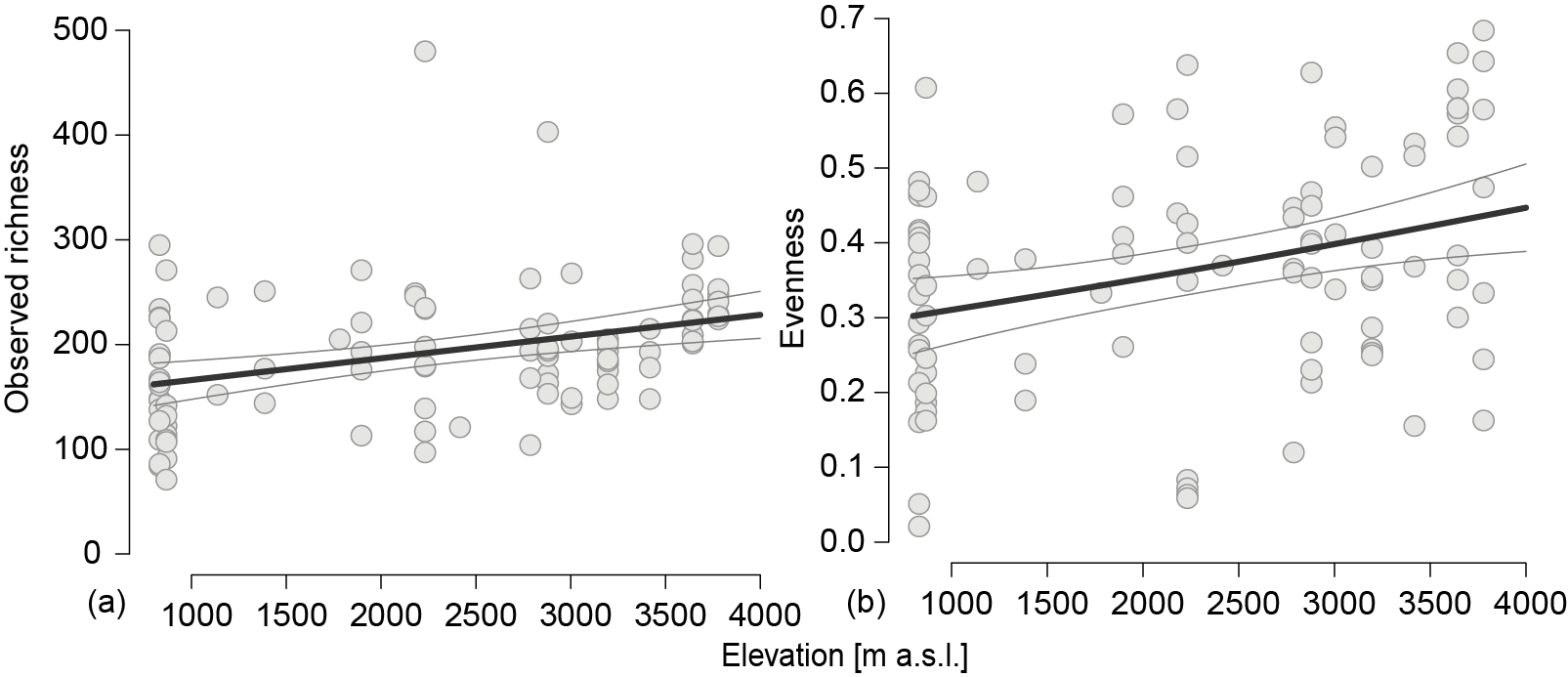 |
| --- |
| Fig. S5.1: Alpha diversity measures of the gut microbiome of *Lasioglossum* bees along the elevational gradient of Mt. Kilimanjaro. Linear models were used to display elevational patterns of observed OTU richness and evenness of OTUs. (a) observed OTU richness (R² = 0.12, p < 0.001) and (b) evenness of OTUs both increase with elevations (R² = 0.09, p < 0.01). Shown are trend lines from model fits with standard deviation. |

| 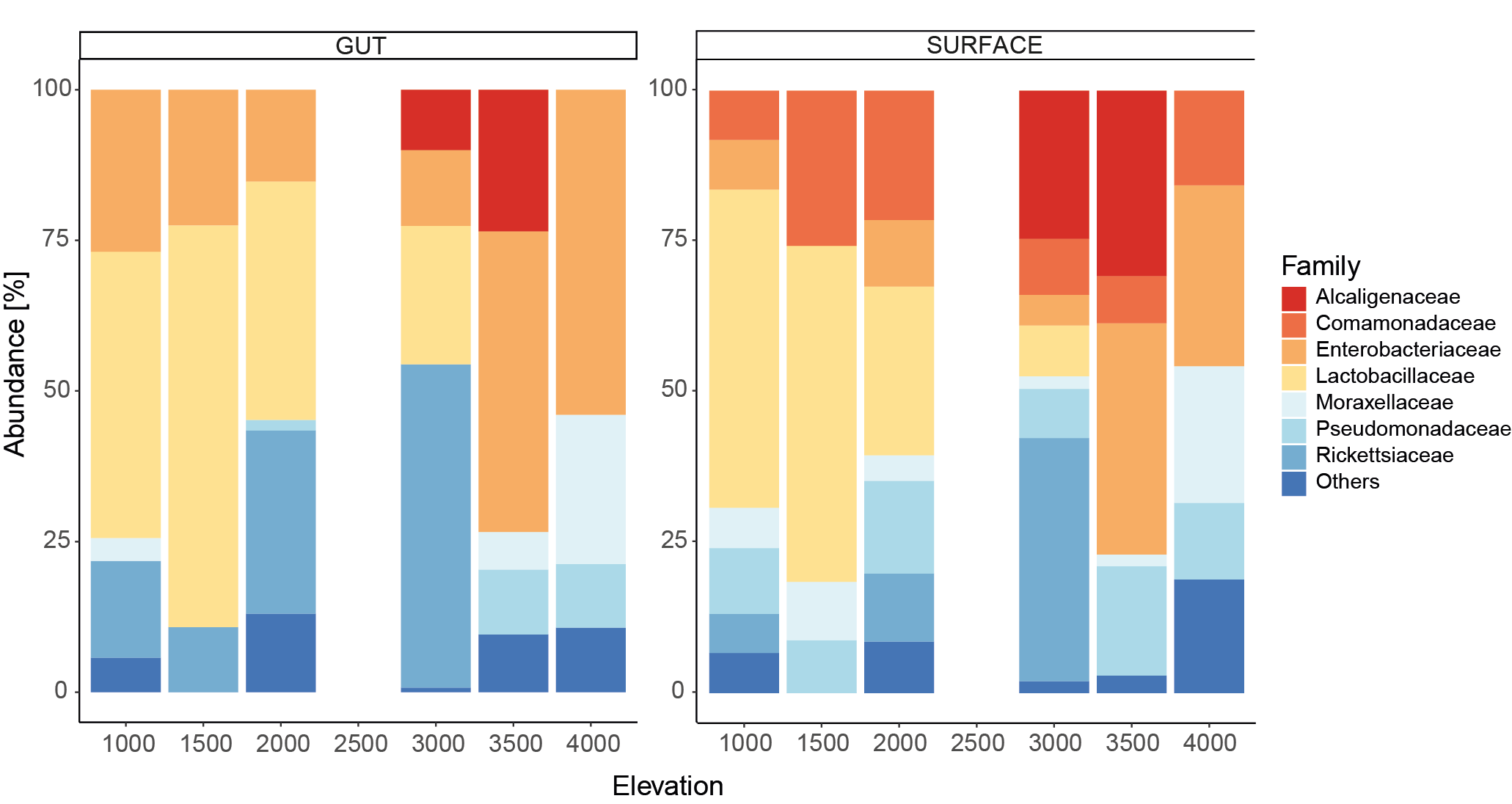 |
| --- |
| Fig. S5.2: Left panel: Gut microbiome and right panel: Surface microbiome of *Lasioglossum* varying on bacterial family level along the elevational gradient, with generally high proportions of Lactobacillaceae, Enterobacteriaceae and Rickettsiaceae. In the surface also Comamonadaceae occur in relatively high proportions along the elevational gradient, which were negligible in gut microbiomes. |

###### Comparative analyses of molecular interactions

| Tab. S6: Best models for cuticular hydrocarbons (CHC), pollen diet, gut and surface microbiome of *Lasioglossum* bees up to Δ AICc < 3 by AICc values. |
| --- |
| \| **Molecular interaction** \| **Axis** \| **NMDS1**  **Pollen** \| **NMDS2**  **Pollen** \| **T** \| **rH** \| **Phylo-species** \| **AICc** \| **Weight** \| **R²** \| **# Sample** \| \| --- \| --- \| --- \| --- \| --- \| --- \| --- \| --- \| --- \| --- \| --- \| \| CHC \| NMDS1 \|  \| -0.28 \| -1.68 \|  \| + \| 14.5 \| 0.29 \| 0.96 \| 28 \| \|  \|  \|  \| -0.28 \|  \| -2.23 \| + \| 14.5 \| 0.29 \|  \|  \| \|  \|  \|  \| -0.28 \|  \| -2.23 \| + \| 14.5 \| 0.29 \|  \|  \| \|  \|  \|  \|  \|  \|  \| + \| 17 \| 0.09 \|  \|  \| \| CHC \| NMDS2 \|  \| 0.13 \|  \| -1.89 \| + \| -11.6 \| 0.24 \| 0.98 \| 28 \| \|  \|  \|  \| 0.13 \|  \| -1.89 \| + \| -11.6 \| 0.24 \|  \|  \| \|  \|  \|  \| 0.13 \| -1.42 \|  \| + \| -11.6 \| 0.24 \|  \|  \| \|  \|  \|  \|  \| -2.11 \|  \| + \| -9.2 \| 2.42 \|  \|  \| \|  \|  \|  \|  \|  \| -2.81 \| + \| -9.2 \| 2.42 \|  \|  \| \|  \|  \|  \|  \|  \| -2.81 \| + \| -9.2 \| 2.42 \|  \|  \| \| **Molecular interaction** \| **Axis** \|  \|  \| **T** \| **rH** \| **Phylo-species** \| **AICc** \| **Weight** \| **R²** \| **# Sample** \| \| pollen \| NMDS1 \|  \|  \| -0.77 \| -0.62 \|  \| 88.9 \| 1.00 \| 0.44 \| 59 \| \| pollen \| NMDS2 \|  \|  \|  \| 0.24 \|  \| 99.5 \| 0.52 \| 0.15 \| 59 \| \|  \|  \|  \|  \| 0.17 \| 0.38 \|  \| 99.8 \| 0.46 \|  \|  \| \| **Molecular interaction** \| **Axis** \| **NMDS1**  **Pollen** \| **NMDS2**  **Pollen** \| **T** \| **rH** \| **Phylo-species** \| **AICc** \| **Weight** \| **R²** \| **# Sample** \| \| gut microbiome \| NMDS1 \| -0.26 \| 0.24 \|  \|  \| + \| 81.1 \| 0.83 \| 0.81 \| 59 \| \| gut microbiome \| NMDS2 \|  \|  \|  \| -0.30 \|  \| 123.8 \| 0.29 \| 0.15 \| 59 \| \|  \|  \|  \|  \| -0.21 \| -0.47 \|  \| 124.1 \| 0.24 \|  \|  \| \|  \|  \| 0.07 \|  \|  \| -0.30 \|  \| 125.4 \| 0.13 \|  \|  \| \|  \|  \|  \| -0.02 \|  \| -0.29 \|  \| 126.1 \| 0.09 \|  \|  \| \|  \|  \| -0.02 \|  \| -0.23 \| -0.48 \|  \| 126.5 \| 0.07 \|  \|  \| \|  \|  \|  \| 0.01 \| -0.21 \| -0.47 \|  \| 126.5 \| 0.07 \|  \|  \| \| **Molecular interaction** \| **Axis** \| **NMDS1**  **CHC** \| **NMDS2**  **CHC** \| **T** \| **rH** \| **Phylo-species** \| **AICc** \| **Weight** \| **R²** \| **# Sample** \| \| head microbiome \| NMDS1 \| 0.49 \|  \|  \| -0.43 \|  \| 76.2 \| 0.43 \| 0.30 \| 42 \| \|  \|  \| 0.45 \| -0.08 \|  \| -0.38 \|  \| 78.1 \| 0.17 \|  \|  \| \|  \|  \| 0.48 \|  \| 0.01 \| -0.42 \|  \| 78.8 \| 0.12 \|  \|  \| \|  \|  \| 0.27 \|  \| 0.30 \|  \|  \| 79.2 \| 0.10 \|  \|  \| \| head microbiome \| NMDS2 \|  \|  \|  \|  \|  \| 81.4 \| 0.27 \|  \| 42 \| \|  \|  \|  \| -0.07 \|  \|  \|  \| 83.1 \| 0.12 \|  \|  \| \|  \|  \|  \|  \| 0.07 \|  \|  \| 83.1 \| 0.11 \|  \|  \| \|  \|  \| -0.07 \|  \|  \|  \|  \| 83.1 \| 0.11 \|  \|  \| \|  \|  \|  \|  \|  \| -0.04 \|  \| 83.5 \| 0.09 \|  \|  \| |
| Shown are standardized explanatory variables from best-fit models. Blue colours indicate positive significant values and red values indicate negative significant values from LM models for the best model each. The significances between phylospecies vary, turquoise means that there is at least one phylospecies correlating significantly with the response variable. T: mean annual temperature, rH: mean annual relative humidity, # Samples: number of samples taken into account for the model. |
